# Genetic deletion of *Alx/Fpr2* differentially regulates pulmonary inflammation in the absence and presence of acute lung injury

**DOI:** 10.1093/immhor/vlaf043

**Published:** 2025-09-17

**Authors:** Rafia Virk, Madeline Behee, Abrar Al-Shaer, Megan Wagner, Michael Armstrong, Nichole Reisdorph, Brooke Bathon, Nari Beatty, Traci Davis, Michael J Yaeger, Rosemary S Gray, Meagan D Bridges, Kymberly M Gowdy, Saame Raza Shaikh

**Affiliations:** Department of Nutrition, Gillings School of Global Public Health and School of Medicine, The University of North Carolina at Chapel Hill, Chapel Hill, NC, United States; Department of Nutrition, Gillings School of Global Public Health and School of Medicine, The University of North Carolina at Chapel Hill, Chapel Hill, NC, United States; Department of Nutrition, Gillings School of Global Public Health and School of Medicine, The University of North Carolina at Chapel Hill, Chapel Hill, NC, United States; Department of Nutrition, Gillings School of Global Public Health and School of Medicine, The University of North Carolina at Chapel Hill, Chapel Hill, NC, United States; Department of Pharmaceutical Sciences, University of Colorado Denver Anschutz Medical Campus, Aurora, CO, United States; Department of Pharmaceutical Sciences, University of Colorado Denver Anschutz Medical Campus, Aurora, CO, United States; Department of Nutrition, Gillings School of Global Public Health and School of Medicine, The University of North Carolina at Chapel Hill, Chapel Hill, NC, United States; Department of Nutrition, Gillings School of Global Public Health and School of Medicine, The University of North Carolina at Chapel Hill, Chapel Hill, NC, United States; Department of Nutrition, Gillings School of Global Public Health and School of Medicine, The University of North Carolina at Chapel Hill, Chapel Hill, NC, United States; Division of Pulmonary, Critical Care and Sleep Medicine, The Ohio State University, Columbus, OH, United States; Department of Nutrition, Gillings School of Global Public Health and School of Medicine, The University of North Carolina at Chapel Hill, Chapel Hill, NC, United States; Department of Nutrition, Gillings School of Global Public Health and School of Medicine, The University of North Carolina at Chapel Hill, Chapel Hill, NC, United States; Division of Pulmonary, Critical Care and Sleep Medicine, The Ohio State University, Columbus, OH, United States; Department of Nutrition, Gillings School of Global Public Health and School of Medicine, The University of North Carolina at Chapel Hill, Chapel Hill, NC, United States

**Keywords:** inflammation, lipid mediators, lipopolysaccharide, lung, rodent

## Abstract

The inflammation resolution receptor lipoxin A4/formyl peptide receptor 2 (ALX/FPR2) plays a critical role in immune regulation by binding select oxylipins derived from n-6 and n-3 polyunsaturated fatty acids (PUFAs). While ALX/FPR2 is implicated in controlling inflammation initiation and resolution, its specific role in pulmonary inflammatory responses remains unclear. In this study, we investigated how genetic deletion of *Alx/Fpr2* controls oxylipin levels, immune cell populations, and inflammatory cytokines under conditions of homeostasis and injury. *Alx/Fpr2* knockout (KO) mice exhibited normal food intake and weight gain but showed impaired glucose and lipid metabolism. Targeted lipidomic analyses by liquid chromatography–tandem mass spectrometry revealed elevated pulmonary concentrations of n-6 and n-3 PUFA–derived oxylipins in KO mice compared to controls. Flow cytometry further demonstrated increased lung infiltration of NK cells, monocytes, and lymphoid cells, indicating a proinflammatory state in the absence of injury. Following 24 h of LPS-induced acute lung injury, IL-1β levels were elevated in KO mice, but pulmonary histopathology, immune cell numbers, and oxylipin levels were comparable to those of controls. These results suggested a protective role of ALX/FPR2 upon acute lung injury, which led us to further investigate the role of ALX/FPR2 upon 72 h of lung injury. Indeed, *Alx/Fpr2* KO mice showed reduced bronchoalveolar lavage protein concentration and lower levels of IL-6 and TNF-α. Collectively, these findings demonstrate that ALX/FPR2 deficiency promotes basal pulmonary inflammation but protects against prolonged injury-induced inflammation, highlighting the context-dependent role of this receptor in pulmonary inflammation.

## Introduction

Global mortality rates from diseases related to lung inflammation have increased over the past decade.^1^ In 2020, 3 of the 10 major causes of death in the United States were due to lung diseases. These included severe acute respiratory syndrome coronavirus 2 (SARS-CoV-2) in addition to chronic lower respiratory diseases and influenza/pneumonia, which ranked third, fifth, and ninth, respectively.^2^ These lung diseases are detrimental due to the persistence of inflammation characterized by increased neutrophil infiltration and poor resolution of inflammation, which impacts the lungs’ vital function for gas exchange.^3–6^

Inflammation is regulated, in part, by oxylipins synthesized from n-6 and n-3 polyunsaturated fatty acids (PUFAs). Many oxylipins derived from n-6 PUFAs, linoleic acid (LA) and arachidonic acid (AA), can be proinflammatory and include prostaglandins and hydroxyeicosatetraenoic acids (HETEs), among others. For example, prostaglandins increase blood flow, recruit leukocytes to injury sites, and stimulate cytokine production.^7^ Similarly, HETEs contribute to the initiation of inflammation. For instance, 5-HETE and 12-HETE promote neutrophil chemotaxis,^8^^,^^9^ enhance mucus production, and induce airway contraction in lung diseases.^8^^,^^10–12^ A clinical study reported that serum levels of n-6 oxylipins are elevated in patients with allergic bronchopulmonary aspergillosis compared to healthy controls.^11^ In contrast, oxylipins derived from n-3 PUFAs, eicosapentaenoic acid and docosahexaenoic acid, are generally anti-inflammatory and associated with resolving inflammation. These include hydroxydocosahexaenoic acids (HDHAs) and hydroxyeicosapentaenoic acids (HEPEs) and their downstream metabolites, which can resolve inflammation by limiting neutrophil accumulation, inhibiting proinflammatory cytokines, and enhancing phagocytosis and efferocytosis.^13^^,^^14^

A key mechanism regulating inflammation is the activation of the G-protein coupled receptor ALX/FPR2, which binds both the n–6 PUFA–derived anti-inflammatory oxylipin lipoxin A_4_ and the n–3 PUFA–derived oxylipin resolvin D1 (RvD1).^15–20^ The ALX/FPR2 receptor is ubiquitously expressed throughout the body with high levels of expression on immune cells such as macrophages, monocytes, and neutrophils.^20–25^ Engagement of ALX/FPR2 by RvD1 initiates active proresolution signaling, dampening excessive neutrophilic inflammation while promoting anti-inflammatory pathways such as macrophage polarization to reparative phenotypes and clearance of apoptotic cells.^26^^,^^27^ Consistent with its role in resolving inflammation, altered ALX/FPR2 levels have been implicated in disease severity, such as for patients who have severe asthma.^28^ Similarly, in critically ill patients with coronavirus disease 2019 (COVID-19), circulating levels of RvD1 are profoundly decreased relative to milder cases, correlating with impaired resolution and worse clinical outcomes.^29^ In contrast, activation of ALX/FPR2 signaling has demonstrated therapeutic benefits such as accelerating the resolution of lung injury,^30^ enhancing alveolar fluid clearance in acute respiratory distress syndrome models,^31^ and even preventing pulmonary fibrosis.^32^ The human relevance of this receptor–lipid mediator axis is evident, making it a promising target in the treatment of pulmonary diseases.

There is some debate regarding the role of ALX/FPR2 in regulating lung injury and inflammation. Two studies on ALX/FPR2 deficiency in lung inflammation have revealed opposing results.^33^^,^^34^ In a mouse model of LPS-induced acute lung injury (ALI), knockout of the ALX/FPR2 receptor reduced oxidative stress, inflammatory responses, and activation of nuclear factor-κB in the lungs.^33^ In contrast, ALI driven by *Streptococcus pneumoniae* increased pulmonary inflammation, bacterial dissemination, and dysregulation in pulmonary function with *Alx/Fpr2* knockout (KO) mice compared to controls.^34^ These contradictory findings warrant further investigation on the role of *Alx/Fpr2* deficiency in controlling lung inflammation.

The objective of this study was to test how the genetic deletion of *Alx/Fpr2* controls biomarkers of pulmonary inflammation. We first assessed the metabolic profile of the *Alx/Fpr2* KO mice prior to lung injury by evaluating glucose tolerance, fasting glucose/insulin, food intake, and whole-body energy expenditure using indirect calorimetry. We then used targeted mass spectrometry to determine how the loss of *Alx/Fpr2* alters the concentrations of n-6 and n-3 PUFA–derived oxylipins in the absence of lung injury. Flow cytometry was employed to quantify key immune cell populations in the lungs. Finally, we examined how the loss of *Alx/Fpr2* controlled oxylipins and immune cell populations in response to LPS-induced ALI at 24 h, which ultimately led to select studies at 72 h post-ALI.

## Materials and methods

### Mice

All animal experiments complied with the National Institutes of Health *Guide for the Care and Use of Laboratory Animals* and were approved by the University of North Carolina at Chapel Hill Institutional Animal Care and Use Committee. Mice were euthanized by isoflurane inhalation followed by cervical dislocation or exsanguination. Wild-type (WT) C57BL/6J mice were purchased from the Jackson Laboratory (Bar Harbor, ME, USA) for breeding. Additional details on *Alx/Fpr2* KO are below. Both WT and KO mice were bred in-house and age-matched at 6 wk, at which point they were placed on an purified diet (10% kcal from fat, diet formula: D12450B) from Research Diets (New Brunswick, NJ, USA) for 15 wk. Additionally, only homozygous WT and KO male mice for *Alx/Fpr2* were used at age 21–22 wk. The data from the control mice used for the metabolic profiling and targeted lipidomic analysis described below are the same from a previously published study comparing lean and obese mice.^35^

### Generation of an Fpr2 mutant allele by CRISPR/Cas9-mediated genome editing

Whole body *Alx/Fpr2* KO mice were generated using CRISPR/Cas9-genome editing technology on a pure C57BL/6J background as previously described in detail.^36^^,^^37^ Animals were continuously bred in-house, and each generation was genotyped by PCR followed by gel electrophoresis using the primers Fpr2-3ScF1 (5′-TTCTGCCTTCCTTACCTTATGC-3′), Fpr2-3ScR1 (5′-GCAAATGCGTATGAGTATAAATGC-3′), and Fpr2-Del-F1 (5′-CTGTGAAAATGCTCTCCTGTATCA-3′). The assay produced a 238 bp band for the Fpr2 deletion allele and a 433 bp band for the WT allele, with heterozygotes displaying both bands. A representative agarose gel is presented in Fig. S1.

### Metabolic profiling

All metabolic profiling experiments were performed at 19 to 20 wk of age for both WT and KO mice. For the intraperitoneal glucose tolerance test, mice were fasted for 5 h and then intraperitoneally administered 2.5 g of dextrose (Sigma‐Aldrich, St Louis, MO, USA) per kilogram lean body mass.^35^ Postprandial glucose measurements were obtained via a glucometer at 15- to 30-min intervals for the next 1.5 h. Fasting insulin values were measured after a 5-h fast and quantified via ELISA (Crystal Chem, Elk Grove Village, IL, USA). Lean and fat body mass was determined via Echo-MRI as previously described.^38^ Metabolic activity was assessed via indirect calorimetry using metabolic cages as previously shown.^38^

### Targeted lipidomics via liquid chromatography–tandem mass spectrometry

Left lungs were harvested, snap-frozen in liquid nitrogen, and stored at −80°C until they were used for PUFA–derived oxylipin analysis using a targeted mass spectrometry approach. Extensive details of the methods are described elsewhere.^35^^,^^39^ In brief, lung samples in cold methanol (−20°C) were homogenized via Qiagen Tissuelyser LT homogenizer (Qiagen, Germantown, MD, USA) and centrifuged at 4 °C and 14,000 rpm for 10 min. The supernatants were extracted and spiked with an internal standard solution (Cayman Chemical, Ann Arbor, MI, USA) before it was dried at 55 °C under vacuum and reconstituted to 10% methanol by volume. Next, the samples were eluted through Strata‐X 33‐μm 30 mg/1 mL solid phase extraction columns (Phenomenex, Torrance, CA, USA) using one volume of methyl formate and methanol, which were both completely dried under nitrogen gas. The subsequent residue was reconstituted in 20 μL ethanol and analyzed via 2‐dimensional reverse phase HPLC tandem mass spectrometry (liquid chromatography–tandem mass spectrometry [LC-MS/MS]) containing an EclipsePlusC18 150 mm analytical HPLC column (Agilent Technologies, Santa Clara, CA, USA). The parameters for LC-MS/MS and specific internal standards are the same as previously described.^35^ Note that our current liquid chromatography method was not able to fully separate the 14-HDHA and 11-HDHA standards for lungs that were treated with LPS, so they were measured together as previously shown.^40^ While other methods have separated these isomers in past studies, our pilot experiments under the same conditions did not detect any 11-HDHA. Therefore, we can conclude that most of the combined signal comes from 14-HDHA.

### Flow cytometry

Harvested lungs were perfused by nicking the left atrium and injecting 10 mL of 1× PBS into the right ventricle using a 20 G 1 1/4′′ needle. The lungs were inflated through the trachea with 3 to 5 mL of digest buffer, consisting of 1.5 mg/mL collagenase A (Sigma-Aldrich, 10103586001) and 0.4 mg/mL DNase (Sigma-Aldrich, 10104159001) in 1× HBSS supplemented with 5% FBS and 10 mM HEPES. Inflated lungs were excised and placed in a 50-mL tube containing 5 mL of additional digest buffer, then incubated in a 37 °C water bath for 40 min, with vortexing every 8 to 10 min. The reaction was stopped by adding 20 mL of cold 1× PBS, and the resulting solution was filtered through a 70-µm cell strainer. The solution was centrifuged at 300 × *g* for 5 min at 4 °C, and RBCs were lysed with 3 mL of ACK RBC lysis buffer (0.17 M NH4Cl, 10 mM KHCO_3_, 250 µM EDTA in DiH_2_O, pH 7.5) for 3 min. The resulting single-cell suspension was pelleted and resuspended in 1× PBS for cell counting. The cells were then resuspended in FACS buffer (3% FBS in 1× PBS) and plated at 4 million cells/250 µL in a 96-well U-bottom plate. Plated cells were stained for dead cells using Zombie Aqua Fixable Viability Dye (BioLegend, 423102) for 15 min with shaking in the dark at 4 °C, followed by 2 washes with FACS buffer. For antibody staining, cells were first blocked with a 1:100 dilution of FcR blocking solution (Miltenyi Biotec, 130092575) and a 1:20 dilution of True-Stain Monocyte Blocking Solution (BioLegend, 426102) for 10 min with shaking in the dark at 4 °C. After one wash, cells were stained with an antibody cocktail consisting of 9 fluorescent antibody markers (Table 1) for 20 min with shaking in the dark at 4 °C. Cells were then washed twice with FACS buffer and incubated with 4% paraformaldehyde for 15 min at room temperature in the dark. After 2 additional washes, cells were resuspended in FACS buffer for flow cytometry analysis. Analysis was performed on the Cytek Aurora system, and data analysis was conducted using FlowJo software, following the gating strategy shown in Fig. S2.

**Table 1.** Fluorescent antibodies for flow cytometry.

Antibody	Fluorophore	Dilution	Isotype	Vendor	Catalog no.
**CD11b**	BUV395	1:100	DA/HA IgG2b, κ	BD Biosciences	563553
**Ly6C**	BV421	1:400	Rat IgG2c, κ	BioLegend	128032
**CD24**	BV605	1:100	Rat IgG2b, κ	BioLegend	101827
**Ly6G**	AF488	1:400	Rat IgG2a, κ	BioLegend	127626
**CD64**	PE	1:100	Rat IgG2a, κ	BioLegend	161004
**MHCII**	PE-Cy7	1:5,000	Rat IgG2b, κ	BioLegend	107630
**Siglec F**	APC	1:200	Rat IgG2a, κ	BioLegend	155508
**CD45**	AF700	1:400	Rat IgG2b, λ	BioLegend	147716
**CD11c**	APC/Fire 750	1:200	Armenian Hamster IgG	BioLegend	117352

### Lung histology

Murine lungs were inflated by 10% neutral buffered formalin at a pressure of 25 cm H_2_O through a catheter into the trachea. The lung tissues were then fixed in 10% neutral buffered formalin for 48 h and subsequently dehydrated in 70% ethanol. The tissues were embedded in paraffin and sectioned at 5 µm. The lung sections were stained with H&E. Slides were scanned at 10× brightfield using Versa 200 (Leica Biosystems, Wetzlar, Germany) and read using Aperio ImageScope 12.4.6 software by an experimenter who was blinded to the study design. Lung sections were scored on a scale of 0 to 3, with 3 being the highest level of lung injury. The scoring is based on methods in our previously published study.^41^

### LPS-induced acute lung injury model

LPS from *Escherichia coli* O111: B4 (L2630, Sigma-Aldrich, St Louis, MO, USA) was used to induce acute ALI in both WT and KO mice via oropharyngeal aspiration. Mice were first anesthetized using an isoflurane vaporizer with an oxygen flow rate of 0.8–1 L/min and an isoflurane percentage of 4.5–5. Mice were then secured in place on an incubation stand and administered 50 µL of 1 mg/mL LPS into its mouth while the tongue was extended, and the nostrils were covered. The tongue was extended, and the nostrils were covered for an additional 20–30 breaths, allowing the mouse to breathe in the LPS directly into its lungs.

### Bronchoalveolar lavage fluid collection and analyses

Bronchoalveolar lavage fluid (BALF) was collected immediately following mouse euthanization. All lung lobes were lavaged twice with 1-mL volumes of 1× PBS with protease inhibitor and collected separately as 2 fractions in microcentrifuge tubes. The stock PBS solution for BALF was prepared with 1 tablet of Roche complete mini protease inhibitor cocktail (Sigma Aldrich, 11836153001) in 10 mL PBS. The collected BALF was centrifuged (400 × *g*, 10 min, 4 °C) to pellet cells. The supernatant from the first BALF fraction was used for total protein analysis via a Pierce BCA Protein Assay Kit (ThermoFisher, 23227) and for cytokine analyses of IL-6 (BioLegend, 431304), IL-1β (BioLegend, 432604), and TNF-α (BioLegend, 430904) via ELISA.

### Statistical analyses

Data were analyzed for parametric distributions as previously described^42^ and are expressed as mean ± SD. A 2-way ANOVA with Šídák multiple comparisons test was used to analyze data from the glucose tolerance test. A one-way ANOVA was used with Šídák multiple comparisons test to analyze data for total protein and cytokines in BALF. Outliers in the quantitative RT-PCR data were identified and removed using the Grubbs test (α = 0.05). All other data sets were analyzed via unpaired 2-tailed Student *t*-tests. All statistical analyses were performed in GraphPad Prism version 9.3.0 software. A *P* value <0.05 was considered significant.

## Results

### Genetic deletion of *Alx/Fpr2* leads to dysregulation of glucose and lipid metabolism

**Figure 1.**
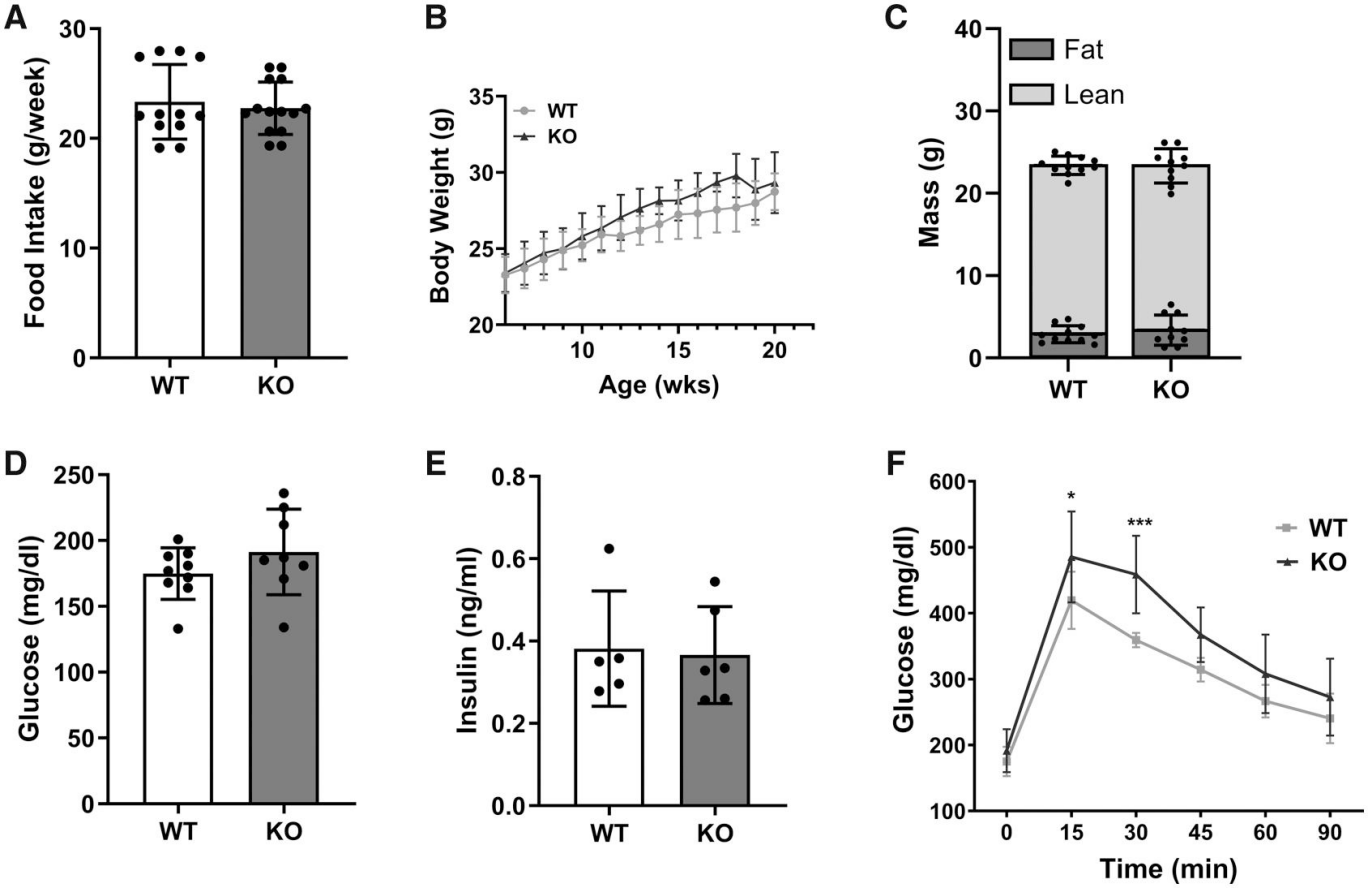
The loss of ALX/FPR2 leads to glucose intolerance. (A) Food intake and (B) body weight as a function of age for WT and *Alx/Fpr2* KO mice. (C) Fat mass and lean mass obtained by Echo-MRI. (D) Fasting glucose and (E) fasting insulin levels obtained after a 5-h fast. (F) The glucose tolerance test was performed after a 5-h fast by intraperitoneal injection of glucose. Metabolic characterization was conducted at 20–21 wk of age. Data are mean ± SD (*n* = 10–14 per group for A–C, *n* = 8–9 per group for D and F, and *n* = 5–6 per group or E). *P < 0.05, ***P < 0.001, from 2-way ANOVA with Šídák multiple comparisons test for (F).

We first characterized the metabolic state of *Alx/Fpr2* KO mice relative to WT mice. There was no change between the WT and KO mice for food intake (Fig. 1A), body weight as a function of age (Fig. 1B), fat and lean mass (Fig. 1C), fasting glucose (Fig. 1D), and fasting insulin (Fig. 1E). The loss of ALX/FPR2 led to increased glucose levels during a glucose tolerance test at 15 and 30 min (Fig. 1F) for *Alx/Fpr2* KO mice relative to WT controls.

**Figure 2.**
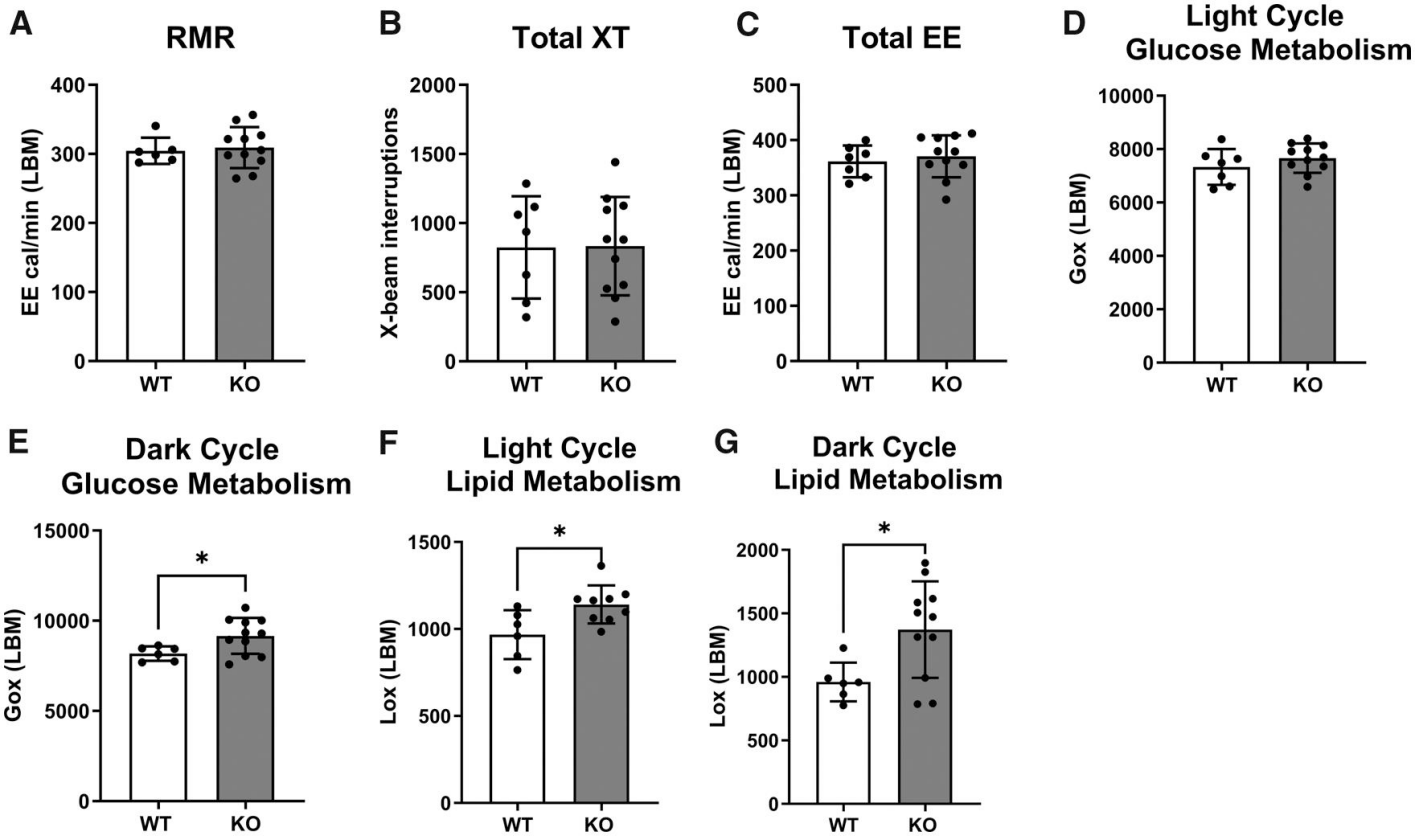
The loss of ALX/FPR2 dysregulates whole body glucose and lipid metabolism. (A) Resting metabolic rate (RMR), (B) total activity (XT), (C) total energy expenditure (EE), (D) light cycle glucose metabolism, (E) dark cycle glucose metabolism, (F) light cycle lipid metabolism, and (G) dark cycle lipid metabolism for WT and *Alx/Fpr2* KO mice. Energy metabolism for mice at 20–21 wk of age was assessed via indirect calorimetry. Data are mean ± SD (*n* = 5–11 per group). *P < 0.05 from unpaired *t*-test. Lox = lipid oxidation. Gox = glucose oxidation.

We further employed indirect calorimetry to investigate the role of ALX/FPR2 on whole body metabolism. Indirect calorimetry experiments revealed no changes in resting metabolic rate (Fig. 2A), total activity (Fig. 2B), total energy expenditure (Fig. 2C), and light cycle glucose metabolism (Fig. 2D) in *Alx/Fpr2* KO relative to WT mice. Interestingly, the dark cycle glucose metabolism (Fig. 2E), light cycle lipid metabolism (Fig. 2F), and dark cycle lipid metabolism (Fig. 2G) were increased in KO mice compared to WT controls. Overall, these findings revealed that the loss of *Alx/Fpr2* dysregulated whole body glucose and lipid metabolism.

### Select pulmonary PUFA–derived oxylipin markers of inflammation are increased in *Alx/Fpr2* KO mice

**Figure 3.**
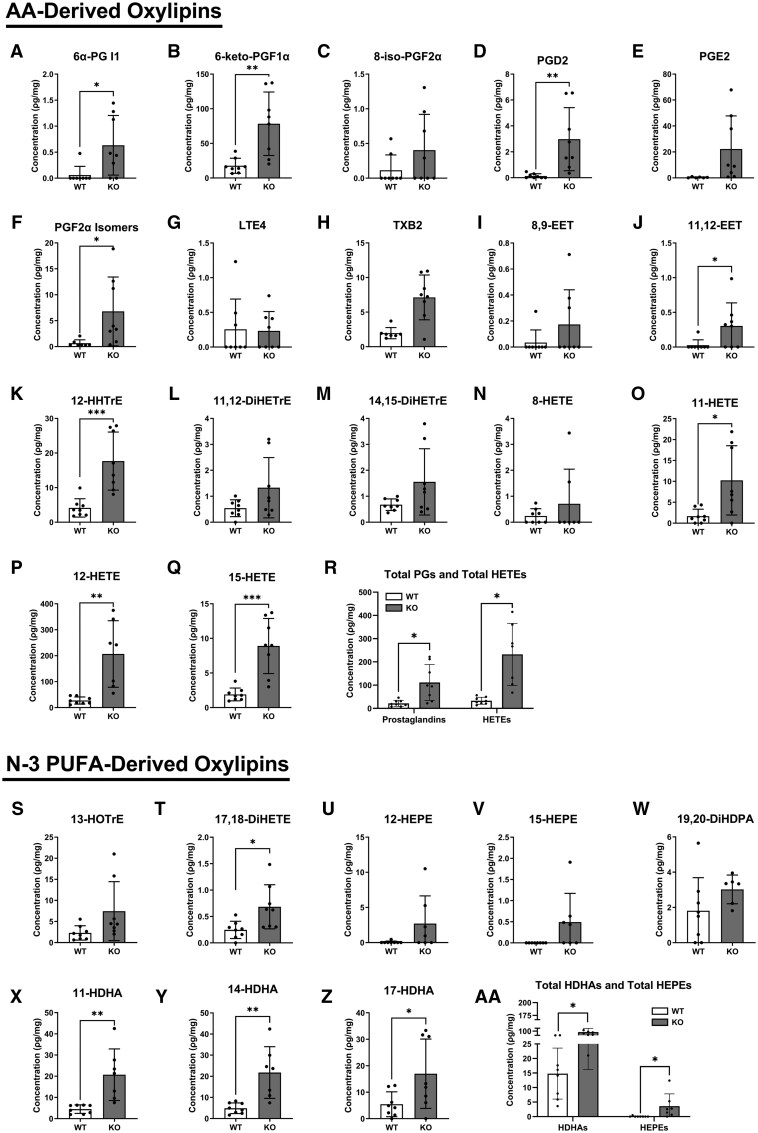
The concentration of select AA-derived and n-3 PUFA–derived oxylipins is increased with the loss of *Alx/Fpr2*. The AA-derived oxylipins are (A) 6α-prostaglandin (PG) I1, (B) 6-keto-PGF1α, (C) 8-iso-PGF2α, (D) PGD2, (E) PGE2, (F) PGF2α isomers, (G) leukotriene E4 (LTE4), (H) thromboxane B2 (TXB2), (I) 8,9-epoxyeicosatrienoic acid (8,9-EET), (J) 11,12-epoxyeicosatrienoic acid (11,12-EET), (K) 12-hydroxyheptadecatrienoic acid (HHTrE), (L) 11,12-dihydroxyeicosatrienoic acid (11,12-DiHETrE), (M) 14,15-dihydroxyeicosatrienoic acid (14,15-DiHETrE), (N) 8-hydroxyeicosatetraenoic acid (8-HETE), (O) 11-hydroxyeicosatetraenoic acid (11-HETE), (P) 12-hydroxyeicosatetraenoic acid (12-HETE), (Q) 15-hydroxyeicosatetraenoic acid (15-HETE), (R) total PGs and total HETEs for WT and *Alx/Fpr2* KO. The select n-3 PUFA–derived oxylipins are (S) 13-hydroxyoctadecatrienoic acid (13-HOTRE), (T) 17,18-hydroxyeicosatetraenoic acid (17,18-DiHETE), (U) 12-hydroxyeicosapentaenoic acid (12-HEPE), (V) 15-hydroxyeicosapentaenoic acid (15-HEPE), (W) 19,20-dihydroxydocosapentaenoic acid (19,20-DiHDPA) (X) 11-hydroxydocosahexaenoic acid (11-HDHA), (Y) 14-hydroxydocosahexaenoic acid (14-HDHA), (Z) 17-hydroxydocosahexaenoic acid (17-HDHA), and (AA) total HDHAs and total HEPEs for WT and *Alx/Fpr2* KO mice. Left lungs from mice aged 21–22 wk were used for targeted LC-MS/MS analysis. Data are mean ± SD (*n* = 7–8 per group). *P < 0.05, **P < 0.01, and ***P < 0.001 from unpaired *t*-test.

At a molecular level, we investigated how the absence of *Alx/Fpr2* controlled the concentration of LA-derived (Fig. S3A–H), AA-derived (Fig. 3A–R), and n-3 PUFA–derived (Fig. 3S–AA) oxylipins, which serve as key lipid mediators of inflammation. The concentrations of LA-derived oxylipins 12,13-DiHOME (Fig. S3B), 9-HODE (Fig. S3E), and 13-HODE (Fig. S3F) increased by 1.8-fold, 2.1-fold, and 2.9-fold, respectively, in *Alx/Fpr2* KO mice. Additionally, differing pulmonary AA–derived oxylipins were significantly elevated in *Alx/Fpr2* KO animals. Relative to controls, the concentration of prostaglandins 6α-PGl1 (Fig. 3A), 6-keto-PGF1α (Fig. 3B), PGD2 (Fig. 3D), and PGF2α isomers (Fig. 3F) were increased by 10.6-fold, 4.4-fold, 22.6-fold, and 66.5-fold, respectively, in the lungs of *Alx/Fpr2* KO mice. 11,12-EET (Fig. 3J) and 12-HHTrE (Fig. 3K) increased by 11.2-fold and 4.3-fold, respectively, in *Alx/Fpr2* KO mice relative to WT mice. Additionally, 11-HETE (Fig. 3O), 12-HETE (Fig. 3P), and 15-HETE (Fig. 3Q) increased by 6.1-fold, 7.6-fold, and 4.7-fold, respectively, with the loss of the receptor. Overall, total pulmonary prostaglandins and HETEs were elevated by 5.2-fold and 7.1-fold in KO relative to WT mice (Fig. 3R).

Select n-3 PUFA–derived oxylipins were also elevated with the loss of the receptor. Notably, 17,18-DiHETE (Fig. 3T), 11-HDHA (Fig. 3X), 14-HDHA (Fig. 3Y), and 17-HDHA (Fig. 3Z) increased 2.8-fold, 4.6-fold, 4.5-fold, and 3.1-fold, respectively, in *Alx/Fpr2* KO mice compared to WT animals. Overall, the total concentration of HDHAs and HEPEs increased by 5.6-fold and 61.9-fold, respectively, in KO versus WT mice (Fig. 3AA). Together, these results demonstrate that the absence of *Alx/Fpr2* leads to a proinflammatory pulmonary oxylipin profile, characterized by elevated levels of select n-6 PUFA–derived and n-3 PUFA–derived oxylipins.

### Select immune cell populations are increased in the lungs with *Alx/Fpr2* deficiency

**Figure 4.**
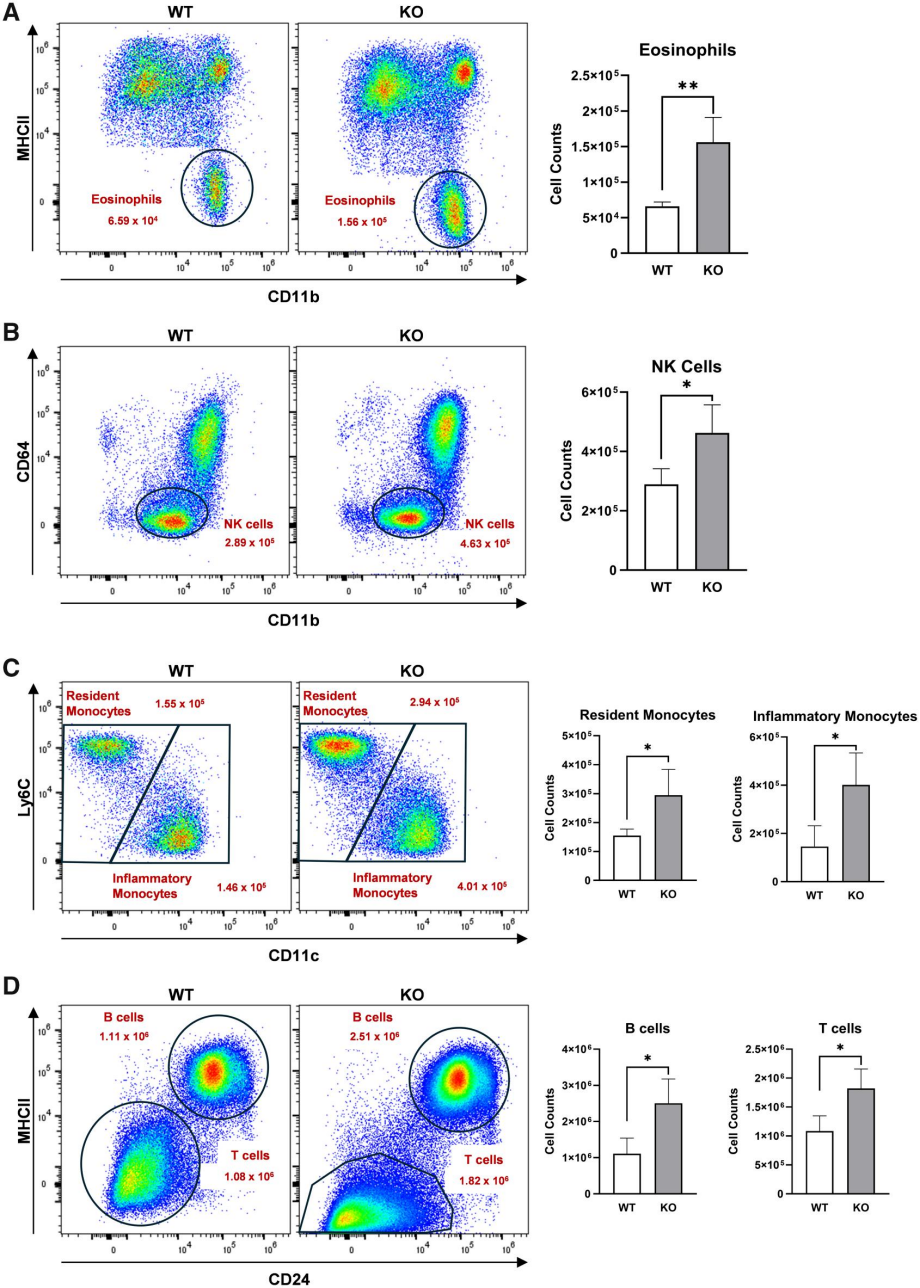
The loss of ALX/FPR2 increases select immune cell populations in the lungs. Lung tissues were harvested, digested into single-cell suspensions, and stained with a panel of fluorescent antibodies. Samples were acquired using the Cytek Aurora spectral flow cytometer and analyzed with FlowJo software. Quantified immune cell populations include (A) eosinophils, (B) NK cells, (C) resident and inflammatory monocytes, and (D) B and T cells. Representative flow cytometry plots and corresponding bar graphs are presented for each population. Data are mean ± SD (*n* = 3–4 per group). *P < 0.05 and **P < 0.01 from unpaired *t*-test.

We subsequently investigated how the loss of *Alx/Fpr2* controlled pulmonary immune cell populations. The analysis revealed that specific immune cell populations increased with the loss of *Alx/Fpr2* (Fig. 4), while others remained unchanged (Fig. S4). Specifically, there was a 2.37-fold increase for eosinophils (Fig 4A), 1.6-fold for NK cells (Fig 4B), 1.9-fold for resident monocytes (Fig 4C), 2.75-fold for inflammatory (recruited) monocytes (Fig 4C), 2.26-fold for B cells (Fig 4D), and 1.69-fold for T cells (Fig 4D) relative to WT controls. The increase in select immune cell populations suggests that, due to *Alx/Fpr2* deficiency, the lungs are inflamed in the absence of injury.

### Following 24 h of LPS-induced ALI, PUFA-derived oxylipin levels in *Alx/Fpr2* KO mice are comparable to those in WT controls

**Figure 5.**
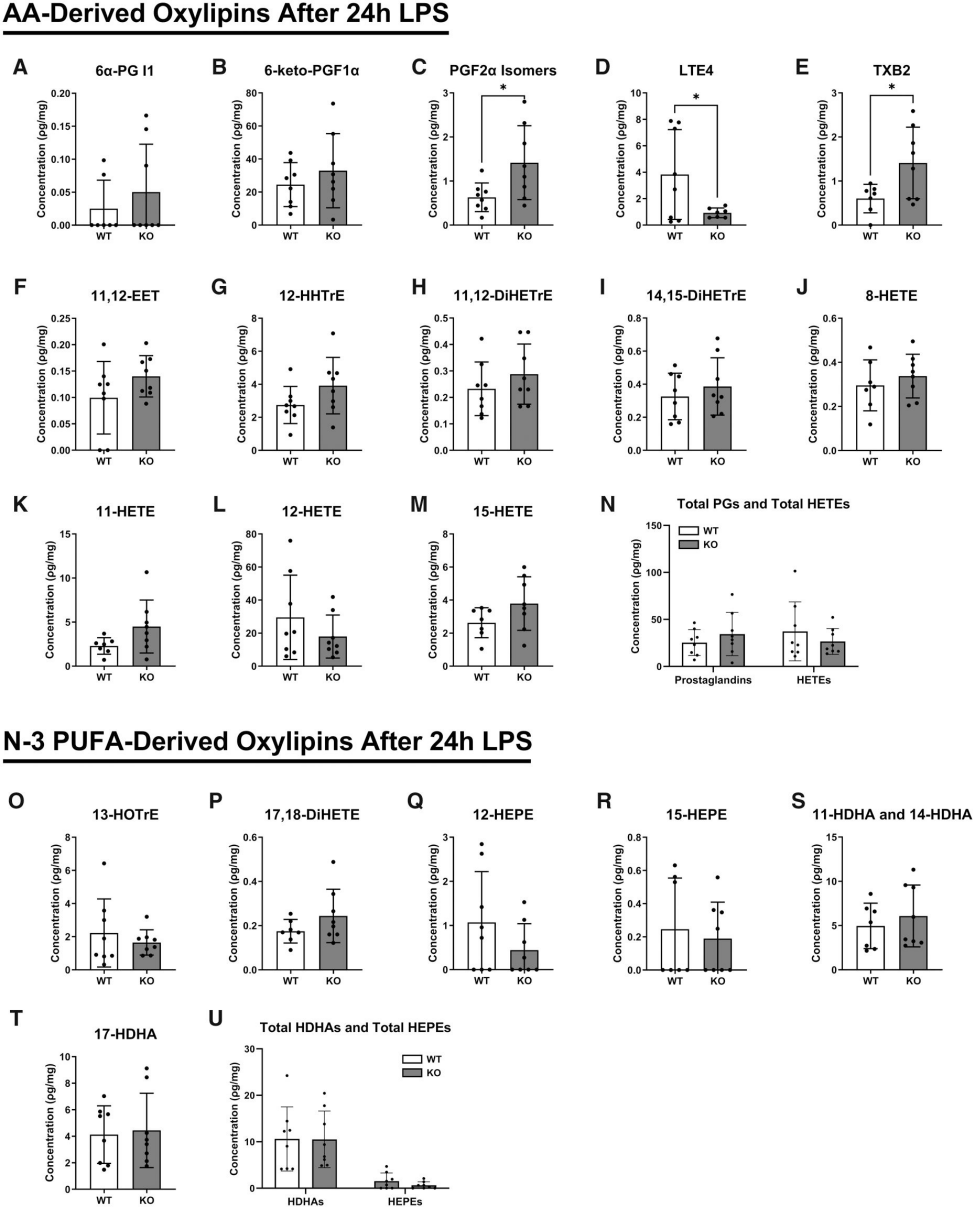
The concentration of select AA-derived and n-3 PUFA–derived oxylipins for *Alx/Fpr2* KO mice is generally normalized to the concentration in WT after 24 h of LPS-induced ALI. The AA-derived oxylipins are (A) 6α-prostaglandin (PG) I1, (B) 6-keto-PGF1α, (C) PGF2α isomers, (D) leukotriene E4 (LTE4), (E) thromboxane B2 (TXB2), (F) 11,12-epoxyeicosatrienoic acid (11,12-EET), (G) 12-hydroxyheptadecatrienoic acid (HHTrE), (H) 11,12-dihydroxyeicosatrienoic acid (11,12-DiHETrE), (I) 14,15-dihydroxyeicosatrienoic acid (14,15-DiHETrE), (J) 8-hydroxyeicosatetraenoic acid (8-HETE), (K) 11-hydroxyeicosatetraenoic acid (11-HETE), (L) 12-hydroxyeicosatetraenoic acid (12-HETE), (M) 15-hydroxyeicosatetraenoic acid (15-HETE), (N) total PGs and total HETEs for WT and *Alx/Fpr2* KO mice. The select n-3 PUFA–derived oxylipins are (O) 13-hydroxyoctadecatrienoic acid (13-HOTRE), (P) 17,18-hydroxyeicosatetraenoic acid (17,18-DiHETE), (Q) 12-hydroxyeicosapentaenoic acid (12-HEPE), (R) 15-hydroxyeicosapentaenoic acid (15-HEPE), (S) 11-hydroxydocosahexaenoic acid (11-HDHA) and 14-hydroxydocosahexaenoic acid (14-HDHA), (T) 17-hydroxydocosahexaenoic acid (17-HDHA), and (U) total HDHAs and total HEPEs for WT and *Alx/Fpr2* KO mice. Left lungs from mice aged 21–22 wk were used for targeted LC-MS/MS analysis. Data are mean ± SD (*n* = 7–8 per group). *P < 0.05 from unpaired *t*-test.

**Figure 6.**
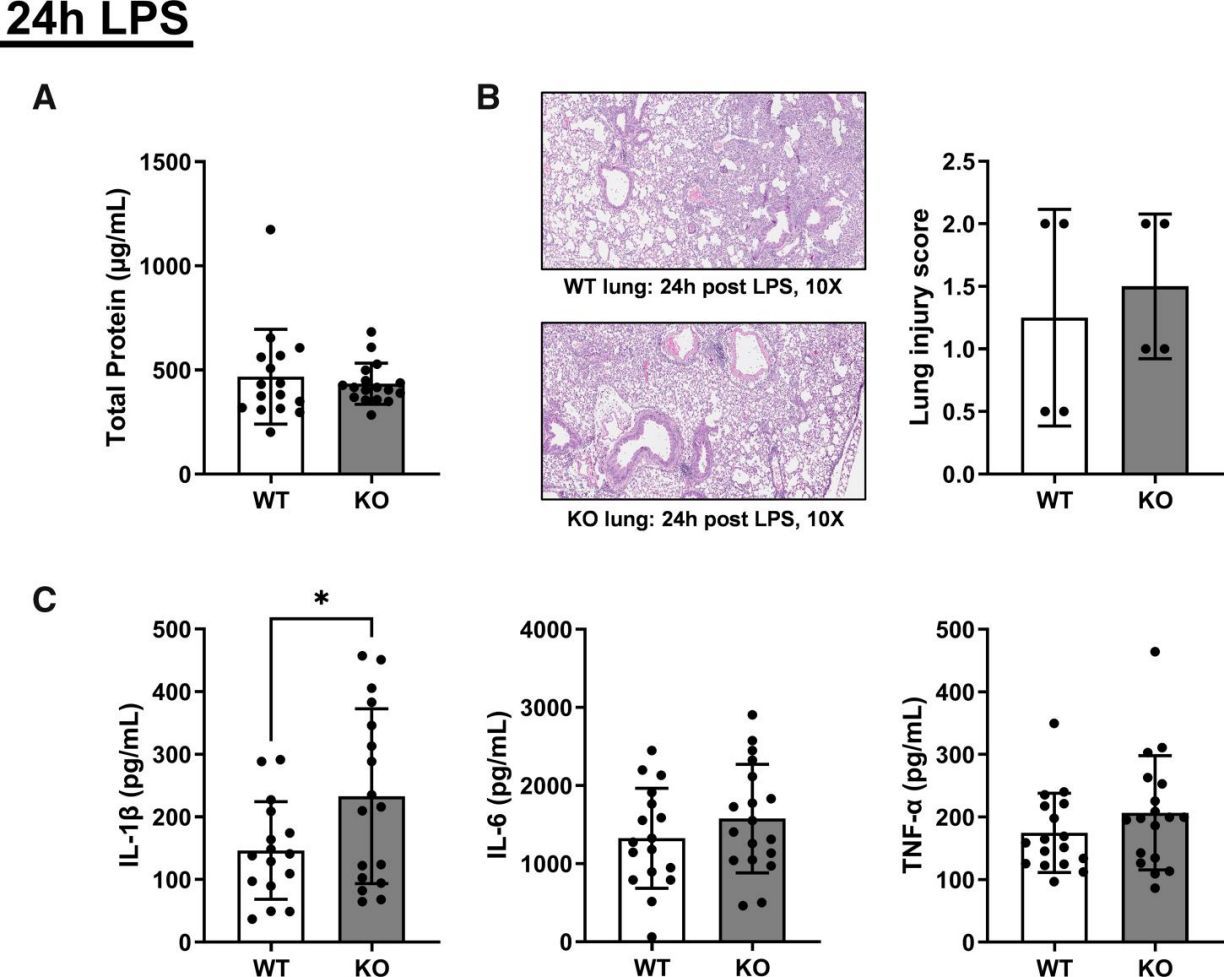
IL-1β levels are increased after 24 h of LPS-induced ALI with the loss of *Alx/Fpr2*. (A) Total protein levels in BALF 24 h after LPS-induced ALI. (B) Images from histological analyses using H&E. Images of lungs are shown in 10× brightfield magnification for WT and *Alx/Fpr2* KO mice 24 h after LPS administration. Lung injury scores were conducted blinded and are on a 0 to 3 scale, with 3 being the highest level of lung injury. (C) IL-1β, IL-6, and TNF-α levels in BALF 24 h after LPS-induced ALI. Data are mean ± SD (*n* = 16–18 per group for A and C and *n* = 4 per group for B). *P < 0.05 from unpaired *t*-test.

To determine whether the preexisting inflammation observed in ALX/FPR2-deficient lungs exacerbated the immune response following ALI, we assessed biomarkers of pulmonary inflammation after 24 h of LPS-induced ALI. We first investigated how ALI controlled the concentration of LA-derived (Fig. S5A–G), AA-derived (Fig. 5A–N), and n-3 PUFA–derived (Fig. 5O–U) oxylipins. The concentrations of the LA-derived oxylipins 12,13-DiHOME (Fig. S6B), 9-HODE (Fig. S6D), and 13-HODE (Fig. S6E) were not increased in *Alx/Fpr2* KOs relative to controls. Additionally, the concentrations of the AA-derived prostaglandins 6α-PGl1 (Fig. 5A) and 6-keto-PGF1α (Fig. 5B) were unchanged relative to WT concentrations. However, PGF2α isomers (Fig. 5C) remained elevated in the lungs of *Alx/Fpr2* KO mice after ALI, with a 2.2-fold increase. LTE_4_ was the only oxylipin that showed an increase in WT mice following LPS treatment, with levels rising 15-fold (Fig. 5D). In contrast, in *Alx/Fpr2* KO mice, LTE4 levels were 4.13-fold lower than in WT mice after ALI (Fig. 5D). The concentration of TXB2 was increased in KO mice by 2.3-fold relative to WT mice (Fig. 5E). Additionally, the oxylipins 11,12-EET (Fig. 5F), 12-HHTrE (Fig. 5G), 11-HETE (Fig. 5K), 12-HETE (Fig. 5L), and 15-HETE (Fig. 5L) were no longer elevated in KOs relative to WT controls. Overall, total pulmonary prostaglandins and HETEs were unchanged in KO relative to WT mice (Fig. 5N) after 24 h of LPS-induced ALI.

The n-3 PUFA–derived oxylipins 17,18-DiHETE (Fig. 5P), 11-HDHA and 14-HDHA (Fig. 5S), and 17-HDHA (Fig. 5T) were also unchanged with the loss of the receptor following 24 h of ALI. Overall, the total concentration of HDHAs and HEPEs were not altered in KO versus WT mice (Fig. 5U). Together, these results demonstrate that by the 24-h timepoint of ALI, the oxylipin profile in KO mice had normalized to that of WT mice.

Following 24 h LPS-induced ALI, the loss of *Alx/Fpr2* increased pulmonary IL-1β levels but did not change immune cell populations. Analysis of BALF after 24 h LPS-induced ALI showed no change in total protein levels, indicative of preserved alveolar–capillary barrier function, as elevated protein levels would typically reflect increased permeability due to lung injury (Fig. 6A). Similarly, blinded analyses of H&E-stained pulmonary tissue revealed no differences in lung injury scores 24 h post–LPS administration between *Alx/Fpr2* KO mice and controls (Fig. 6B).

Compared to controls, ELISA analyses of BALF revealed an increase in the concentration of IL-1β, but not in IL-6 or TNF-α, in *Alx/Fpr2* KO mice after 24 h of ALI (Fig. 6C). We next quantified the number of infiltrating immune cells in the lungs. No differences were observed in the number of any immune cell population in the lungs after 24 h of ALI between *Alx/Fpr2* KO and WT mice (Fig. S5).

### Following 72 h of LPS, *Alx/Fpr2* deficiency protects against ALI

**Figure 7.**
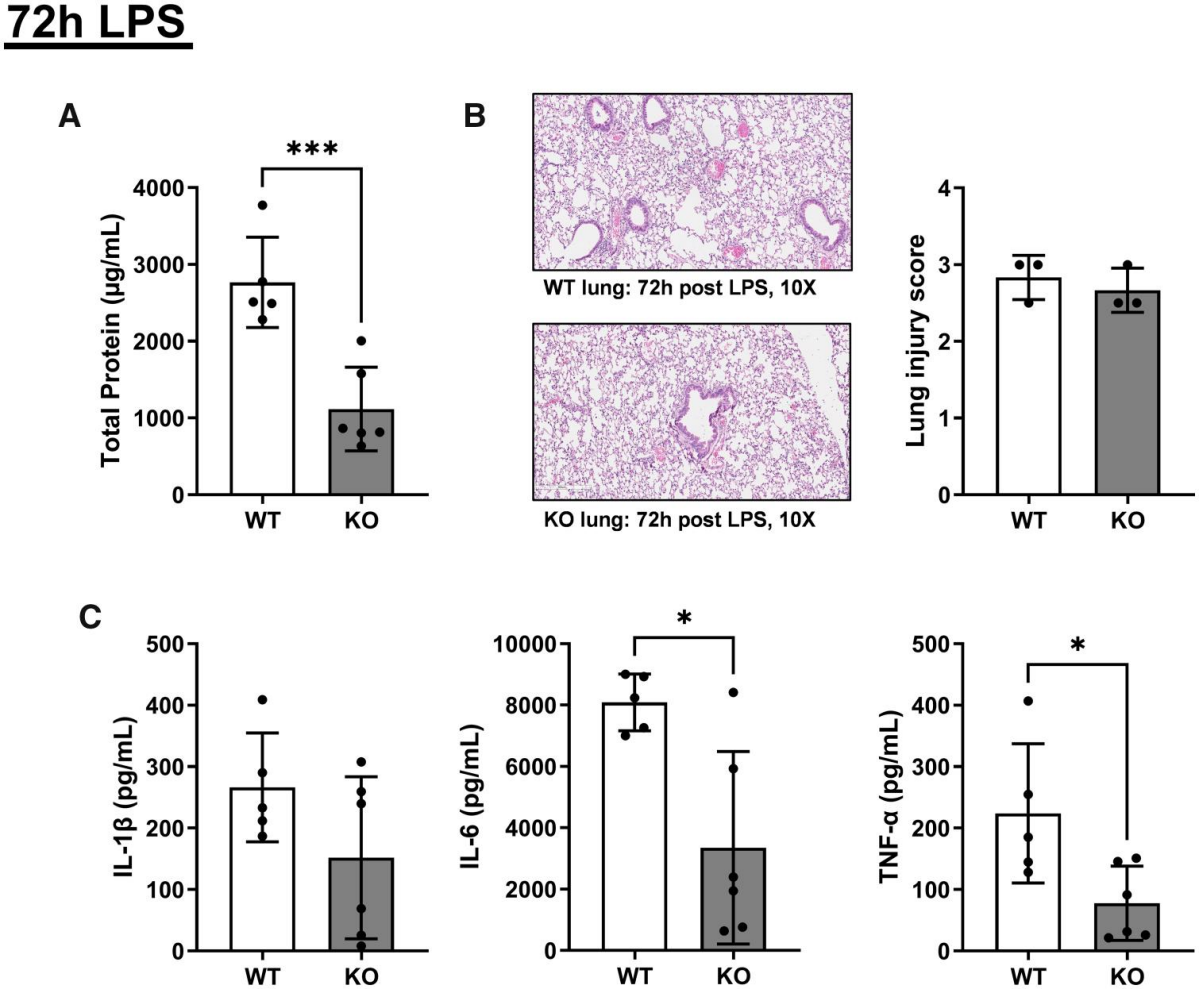
Total protein, IL-6, and TNF-α levels are decreased after 72 h of LPS-induced ALI with the loss of *Alx/Fpr2*. (A) Total protein levels in BALF 72 h after LPS-induced ALI. (B) Images from histological analyses using H&E. Images of lungs are shown in 10× brightfield magnification for WT and *Alx/Fpr2* KO mice 72 h after LPS administration. Lung injury scores were conducted blinded and are on a 0 to 3 scale, with 3 being the highest level of lung injury. (C) IL-1β, IL-6, and TNF-α levels in BALF 72 h after LPS-induced ALI. Data are mean ± SD (*n* = 5–6 per group for A and C and *n* = 3 per group for B). *P < 0.05 and ***P < 0.001 from unpaired *t*-test.

Finally, our results did not align with a previous study that reported improved pulmonary inflammation with the loss of *Alx/Fpr2*.^33^ In addition, our data at 24 h suggested that ALI was not significantly exacerbated by the loss of the receptor. Therefore, we hypothesized that ALX/FPR2 may modulate inflammatory cytokines at a different time point following LPS activation. We specifically investigated pulmonary inflammation markers after 72 h of LPS-induced ALI. Total protein in BALF was reduced by 2.47-fold in KO mice compared to controls (Fig. 7A). Although lung injury scores from histological analysis did not differ (Fig. 7B), levels of IL-6 and TNF-α decreased by 2.42-fold and 2.88-fold, respectively, in KO relative to WT mice (Fig. 7C). Collectively, these findings indicate that at 72 h of ALI, the loss of *Alx/Fpr2* is associated with a reduced inflammatory response.

## Discussion

In this study, we tested the hypothesis that ALX/FPR2, an inflammation resolution receptor, controls aspects of pulmonary inflammation. ALX/FPR2 signaling is mediated by the binding of various ligands including annexin A1,^34^^,^^43^ lipoxin A4,^18^^,^^25^^,^^44^ and RvD1.^16^^,^^45^^,^^46^ However, there are considerable gaps in our knowledge regarding this receptor’s role in inflammatory processes. Previous studies of *Alx/Fpr2* deficiency show an exacerbated inflammatory response with increased injury in the liver,^47^^,^^48^ brain,^49^ and systemic inflammation.^50^ Although many studies show that ALX/FPR2 deficiency increases inflammation, a few studies show that knockout of *Alx/Fpr2* may be beneficial for some outcomes, such as in the brain for improving cognition and detrimental effects of Alzheimer disease in mice.^51^ One study reported no changes in the inflammatory markers TNF-α, CXCL1, and IL-10 in BALF with knockout of *Alx/Fpr2* relative to WT controls.^52^ Importantly, the impact of *Alx/Fpr2* deficiency on pulmonary inflammation is unclear.^33^^,^^34^

We first found that the loss of *Alx/Fpr2* drove glucose intolerance and dysregulation of glucose and lipid oxidation. This is notable, as the mice exhibited signs of metabolic impairments without an increase in fat mass. There is previous research indicating that ALX/FPR2 controls whole body metabolism^53–55^; however, the evidence is conflicting. For example, a previous study showed that *Alx/Fpr2* KO mice at 4 mo of age spontaneously developed obesity driven by weight gain and cardiometabolic dysfunction.^56^^,^^57^ Our findings are in agreement with the metabolic dysregulation observed by this group,^56^^,^^57^ with the exception of weight gain. It is possible that weight gain could have been achieved if our *Alx/Fpr2* KO mice were aged longer. In contrast, another lab reported that a deficiency of ALX/FPR2 improved insulin sensitivity in diet-induced obese mice through increased weight loss.^58^ These contradictory findings may be explained by differences in mouse age and diet. Future studies will need to investigate how ALX/FPR2 signaling intersects with metabolic processes. For instance, ALX/FPR2 redirects glucose flux and facilitates metabolic reprogramming in lung cancer cells.^53^ Conversely, hyperglycemic conditions suppress *Alx/Fpr2* expression, further illustrating the sensitivity of this pathway to metabolic cues.^59^

A key finding from this research is that pulmonary n-6 and n-3 PUFA–derived oxylipins were elevated in *Alx/Fpr2* KO mice prior to external injury. The n-6 PUFA–derived oxylipins are predominantly involved in the initiation of inflammation, implying that the onset of inflammation is occurring in KO animals prior to lung injury. However, what is surprising is that the oxylipins derived from n-3 PUFAs, such as 17-HDHA, were also increased, which are involved in inflammation resolution pathways.^42^^,^^60^^,^^61^ Perhaps the increase in n-3 PUFA–derived oxylipins is a counter-regulatory mechanism to counteract the increase in n-6 PUFA–derived oxylipins. Data also show that n-3 PUFA–derived oxylipins are elevated in certain tissues.^62^ Studies show that an imbalance between n-6 and n-3 PUFA–derived oxylipins can result in prolonged, unresolved inflammation, thereby increasing susceptibility to and severity of additional illnesses.^63^ For instance, the potential for altered oxylipin levels to drive SARS-CoV-2 illness has been extensively explored in recent years, with experiments showing elevated levels of proinflammatory and proresolving oxylipins in the serum^64^ and BALF^65^ of patients with COVID-19. To further support the inflamed state in KO animals prior to ALI, we quantified immune cell populations and observed increased pulmonary eosinophils, resident monocytes, inflammatory monocytes, B cells, and T cells in KOs relative to WT controls. These results are consistent with a study that reported increased proinflammatory macrophages with inactivation of ALX/FPR2 using an antagonist, WRW4.^66^

Lipidomic analyses of lungs after 24 h of ALI revealed normalization of oxylipin levels in KO to those found in WT mice. The concentrations of LA-derived, AA-derived, and n-3 PUFA–derived oxylipins that were changed prior to ALI were now unchanged relative to levels in WT controls after 24 h of ALI. However, PGF2α isomers remained elevated in the lungs of *Alx/Fpr2* KO mice after ALI, showing only a 2.2-fold increase, a magnitude which is still 64.3-fold lower than the levels observed before ALI. Although the concentration of TXB2 was increased in KO mice, the concentration was lower than the TXB2 concentration before ALI in KOs. Collectively, these data suggest that the loss of *Alx/Fpr2* at the 24-h time point had a limited impact on pulmonary inflammation.

We also discovered that LPS-induced lung injury resulted in elevated levels of IL-1β with the loss of ALX/FPR2 relative to WT controls. LPS increases the concentration of cytokines such as IL-1β, IL-6, and TNF-α in the lungs,^67–70^ but only IL-1β was overtly increased with *Alx/Fpr2* deficiency, suggesting that ALX/FPR2 regulates IL-1β secretion. Research with RvD1, which signals through the ALX/FPR2 receptor, suggests that RvD1 inhibits nucleotide-binding oligomerization domain–like receptor protein 3 signaling, which is responsible for IL-1β generation.^46^^,^^71^^,^^72^ Hence, it is possible that with the loss of ALX/FPR2, RvD1 is not able to limit IL-1β production, thus creating some mild to moderate inflammation. Note that we did not detect RvD1 in our lipidomic analyses. In addition, flow cytometry analysis of the immune cell populations after ALI also showed no differences between WT and KO mice. This is consistent with another study that reported after FPR2 inhibition, activation of inflammation with acute heart failure did not increase leukocyte recruitment.^66^

Given that our results differ from those of a previous study,^33^ we pursued a select study at another time point of LPS administration. At the 72 h ALI time point, levels of total protein, IL-6, and TNF-α were significantly decreased in *Alx/Fpr2* KO mice relative to controls. This suggests that *Alx/Fpr2* deficiency is protective against exacerbated ALI induced by 50 µg LPS at the 72-h time point. These results are consistent with those found by Lui et al, in which deficiency of *Alx/Fpr2*, with 15–17.5 µg LPS for 24 h, protected mice against injury.^33^

There are several avenues for future research, which will include the need to study differing ligands in the context of *Alx/Fpr2* deficiency. Some studies indicate that ALX/FPR2 undergoes ligand-dependent conformational changes to mediate either proinflammatory or anti-inflammatory responses.^73^^,^^74^ For instance, ligands like serum amyloid protein A promote inflammation,^75^ whereas ligands like RvD1 promote resolution.^15^ In addition to ligands, the environmental context also influences the response of the ALX/FPR2 receptor. For example, in early sepsis, both lipoxin A4 and the expression of *Alx/Fpr2* are increased to promote inflammation, whereas in late sepsis, lipoxin A4 improves survival by dampening inflammation through ALX/FPR2.^76^ This could explain why we observe opposing effects from the loss of *Alx/Fpr2* in the absence and presence of ALI. Overall, these findings highlight the need to identify optimal conditions and ligands for activating the proresolving ALX/FPR2 receptor to improve pulmonary inflammatory outcomes.

This study has several limitations. First, experiments were only conducted with male mice. However, it is recognized that sex plays a role in inflammation status.^77–82^ We have previously demonstrated that there are strong sex differences in the concentration of oxylipins in the liver, white adipose tissue, spleen, and bone marrow and thus, there are likely differences in the lungs.^61^^,^^83^^,^^84^ Second, we did not explore how age impacts inflammation and metabolic dysfunction with the loss of ALX/FPR2. It is possible that ALX/FPR2 deficiency could drive weight gain and insulin resistance.^57^ Third, we did not examine how the loss of ALX/FPR2 directly increases oxylipin levels or the LPS-induced secretion of cytokines. Subsequent studies will also need to study the role of glucose and lipid metabolism on pulmonary oxylipins.^85^ Another limitation is that ALX/FPR2 is expressed on various cell types. Since we used a whole-body *Alx/Fpr2* KO mouse model, we cannot determine which cell type is responsible for the increases in select pulmonary oxylipins observed prior to ALI in the KOs. This will require subsequent studies in tissue-specific knockouts of *Alx/Fpr2* to determine the cell type and signaling mechanism of action.

In conclusion, these studies advance the field by demonstrating the dual role of ALX/FPR2 in a mouse model, depending on the presence or absence of ALI. *Alx/Fpr2* deficiency leads to dysregulated glucose and lipid metabolism, as well as elevated levels of select oxylipins that contribute to inflammation prior to ALI. However, following ALI, *Alx/Fpr2* deficiency has limited impact on pulmonary inflammation and may even confer protection against exacerbated responses. These findings help resolve discrepancies in the literature on ALX/FPR2 and pulmonary outcomes and lay the groundwork for future studies aimed at targeting this receptor. Overall, our results demonstrate that ALX/FPR2 plays a critical role in specific aspects of pulmonary inflammation.

## Supplementary Material

vlaf043_Supplementary_Data

## Data Availability

Data are available upon request.
