# Supplementary material for: Genetic deletion of Alx/Fpr2 differentially regulates pulmonary inflammation in the absence and presence of acute lung injury
Source: Immunohorizons. 2025 Sep 17;9(10):vlaf043. doi: 10.1093/immhor/vlaf043 (PMC12448816; doi:10.1093/immhor/vlaf043)
Supplement: vlaf043_Supplementary_Data [file vlaf043_supplementary_data.docx]

**SUPPLEMENTAL INFORMATION**

**Title:** Genetic deletion of ALX/FPR2 differentially regulates pulmonary inflammation in the absence and presence of acute lung injury

**Authors:** Rafia Virk, Madeline Behee, Abrar Al-Shaer, Megan Wagner, Michael Armstrong, Nichole Reisdorph, Brooke Bathon, Nari Beatty, Traci Davis, Michael J. Yaeger, Rosemary S. Gray, Meagan D. Bridges, Kymberly M. Gowdy, and Saame Raza Shaikh^1^

**
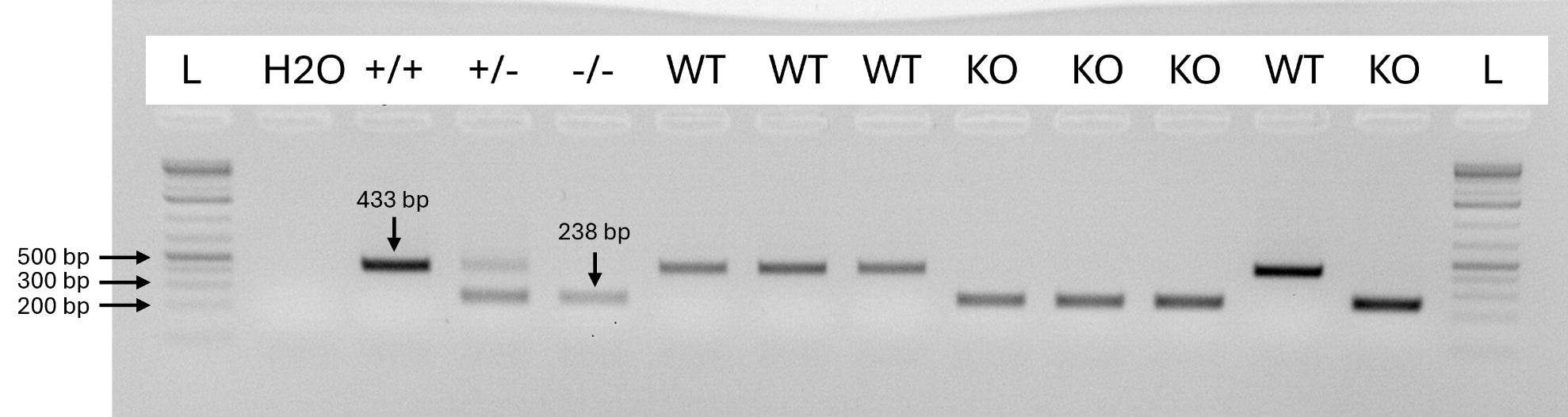
**

**Figure S1: Genotyping of C57B6/J wild-type and FPR2 knockout mice using gel electrophoresis.** Animals were genotyped by PCR followed by gel electrophoresis using the primers Fpr2-3ScF1 (5’-TTCTGCCTTCCTTACCTTATGC-3’), Fpr2-3ScR1 (5’-GCAAATGCGTATGAGTATAAATGC-3’), and Fpr2-Del-F1 (5’-CTGTGAAAATGCTCTCCTGTATCA-3’). The assay produced a 238 bp band for the Fpr2 deletion allele and a 433 bp band for the wild-type allele, with heterozygotes displaying both bands. On the left and right of the gel are the ladder base pairs (L). The controls include water, wild-type (+/+), heterozygous (+/-), and knockout (-/-) alleles. N = 4 for wildtype (WT) and knockout (KO) animals from one experiment.


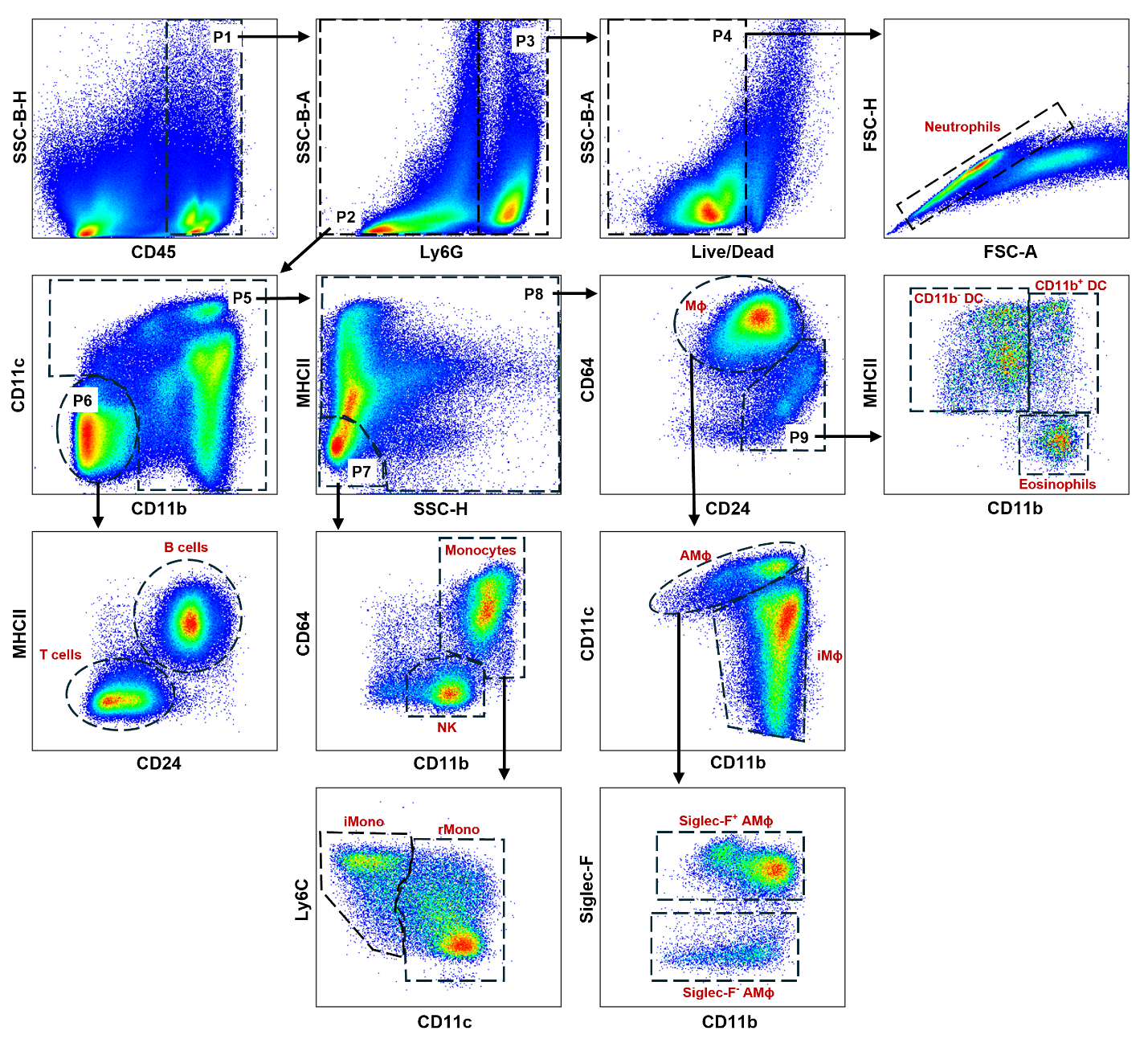


**Figure S2: Gating strategy for lung tissue analysis following 24 h LPS exposure.** Lung cells were processed for flow cytometry, with initial gating to exclude dead cells and doublets across all immune cell populations. For clarity, representative plots illustrating dead cell and doublet exclusion are shown for neutrophils.


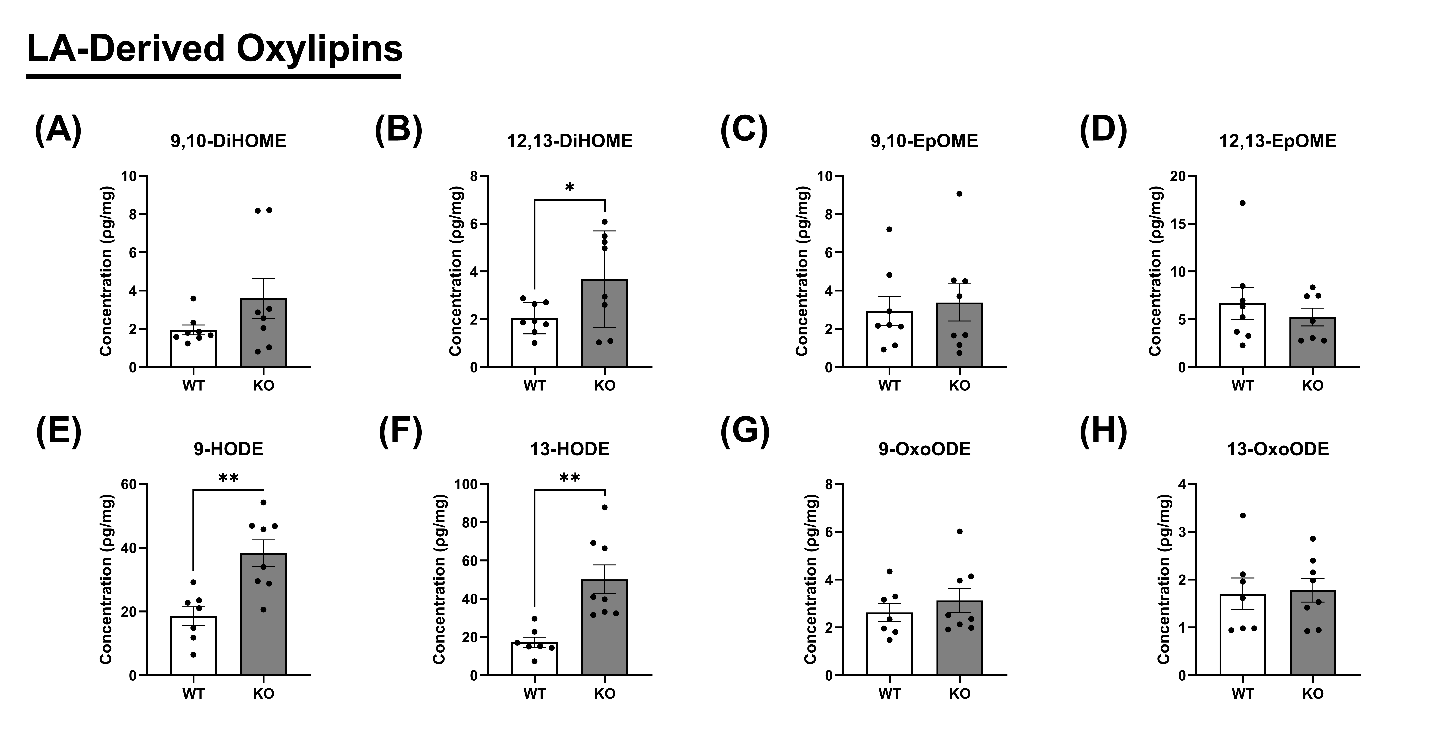


**Figure S3: The concentration of select LA-derived oxylipins is increased with the loss of *Alx/Fpr2*.** The linoleic acid (LA)-derived oxylipins are **(A)** 9,10-dihydroxyoctadecenoic acid (9,10-DiHOME), **(B)** 12,13-dihydroxyoctadecenoic acid (12,13-DiHOME), **(C)** 9,10-epoxyoctadecenoic acid (9,10-EpOME) **(D)** 12,13-epoxyoctadecenoic acid (12,13-EpOME), **(E)** 9-hydroxyoctadecenoic acid (9-HODE), **(F)** 13-hydroxyoctadecenoic acid (13-HODE), **(G)** 13-oxooctadecenoic acid (13-OxoODE), **(H)** 13-oxooctadecenoic acid (13-OxoODE). Left lungs from mice aged 21–22 weeks were used for targeted LC-MS/MS analysis. Data are mean ± SD (n = 7-8) from one experiment. *P < 0.05 and **P <0.01 from an unpaired t-test.


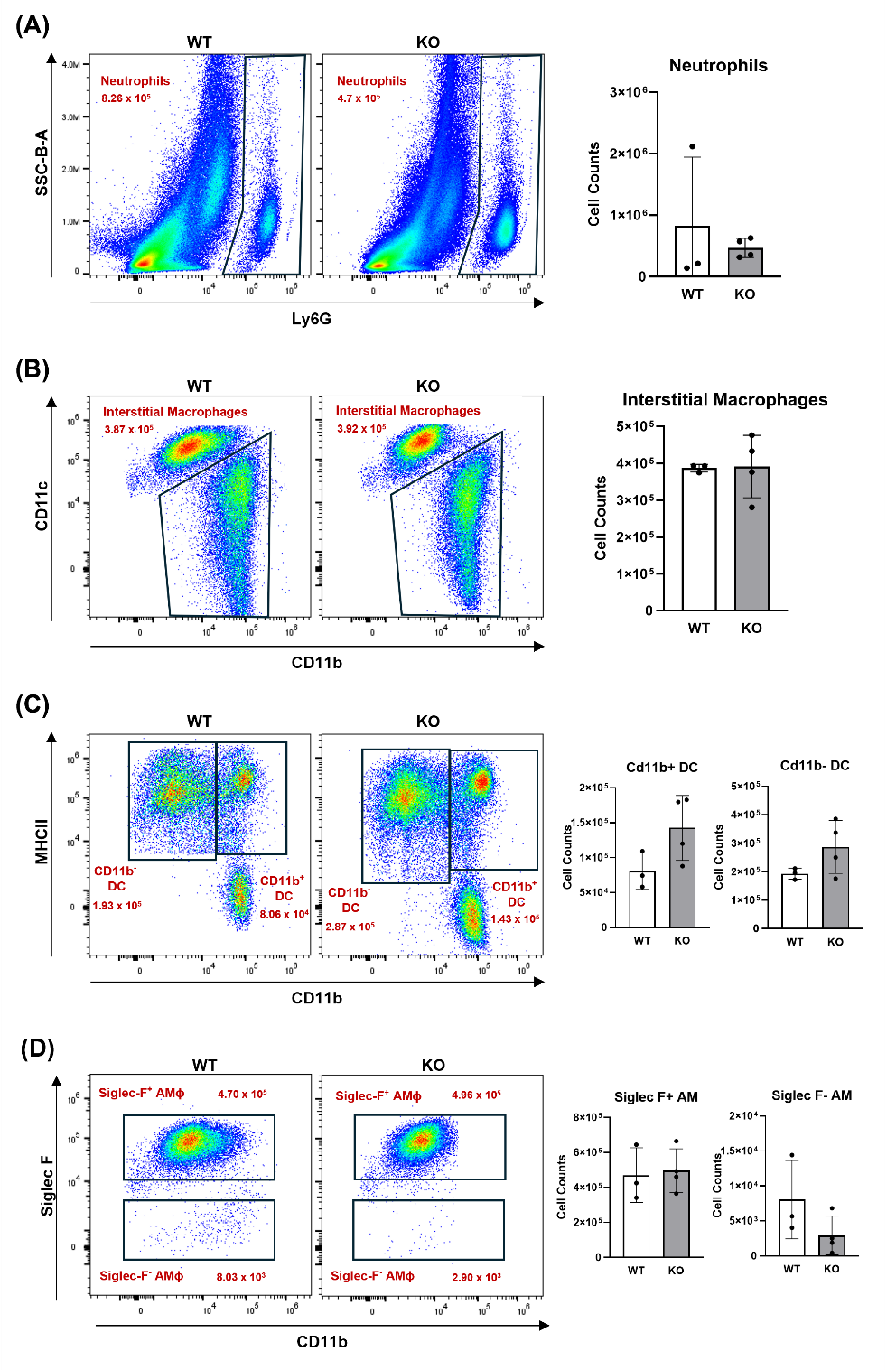


**Figure S4: Immune cell populations in the lungs that were not changed due to loss of *Alx/Fpr2*.** Lung tissues were harvested, digested into single-cell suspensions, and stained with a panel of fluorescent antibodies. Samples were acquired using the Cytek Aurora spectral flow cytometer and analyzed with FlowJo software. Quantified immune cell populations include **(A)** Neutrophils, **(B)** interstitial macrophages, **(C)** CD11b+ and CD11b- dendritic cells (DC), and **(D)** Siglec F+ and Siglec F- alveolar macrophages (AM). Representative flow cytometry plots and corresponding bar graphs are presented for each population. Data are mean ± SD (n = 3–4) from 2 experiments. *P < 0.05, **P < 0.01 from unpaired t-test.


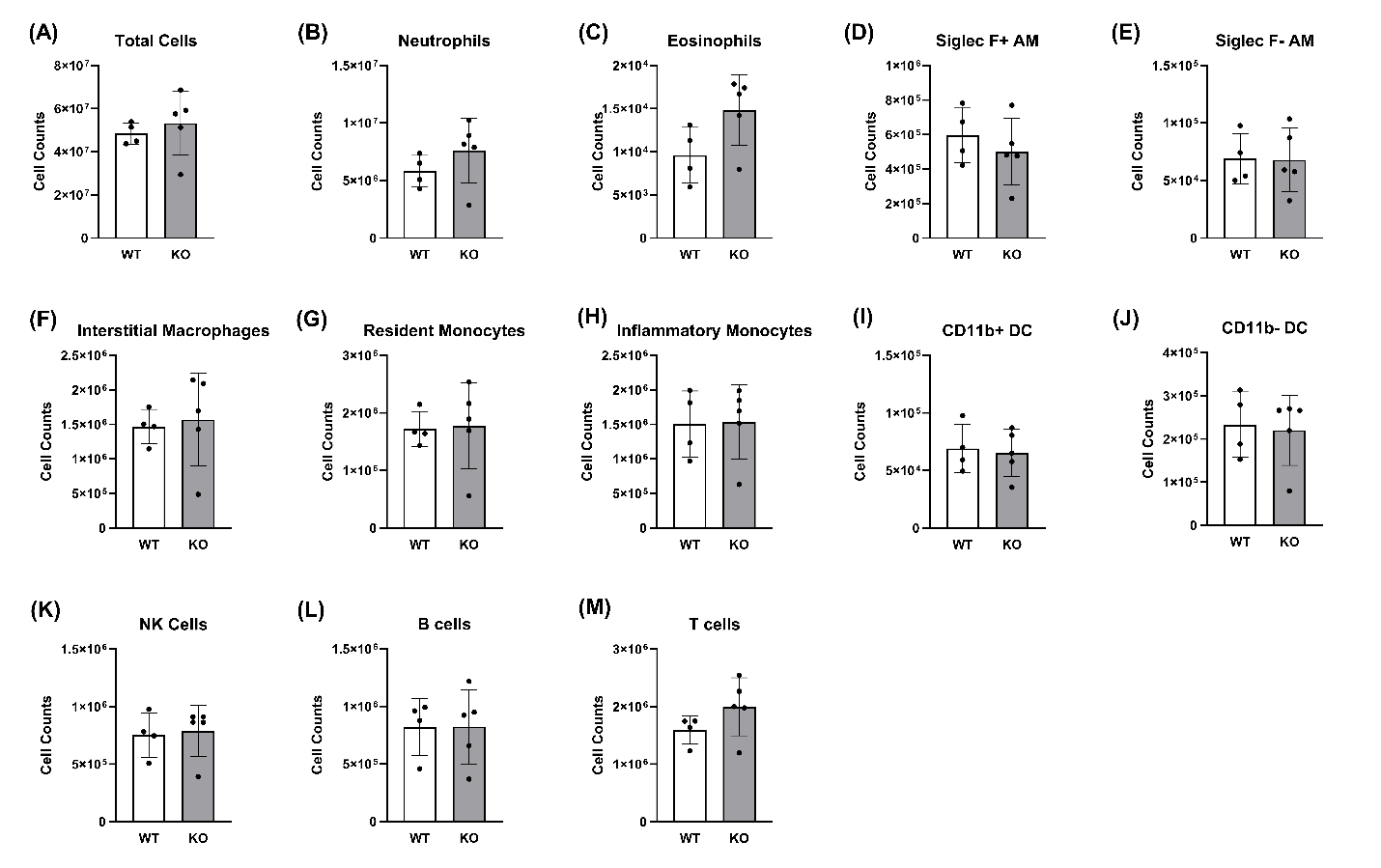


**Figure S5: Flow cytometry analysis of immune cell populations in lungs following 24 h LPS administration.** Lung tissues were harvested, digested into single-cell suspensions, and stained with a panel of fluorescent antibodies. Samples were acquired using the Cytek Aurora spectral flow cytometer and analyzed with FlowJo software. **(A)** Total lung cells, **(B)** neutrophils, **(C)** eosinophils, and **(D)** Siglec F+ alveolar macrophages (AM), **(E)** Siglec F- AM, **(F)** interstitial macrophages, **(G)** resident monocytes, **(H)** inflammatory monocytes, **(I)** CD11b+ dendritic cells (DC), **(J)** CD11b- DC, **(K)** NK cells, **(L)** B cells, and **(M)** T cells. Data are presented as mean ± SD (n = 4–5 per group) from 2 experiments.


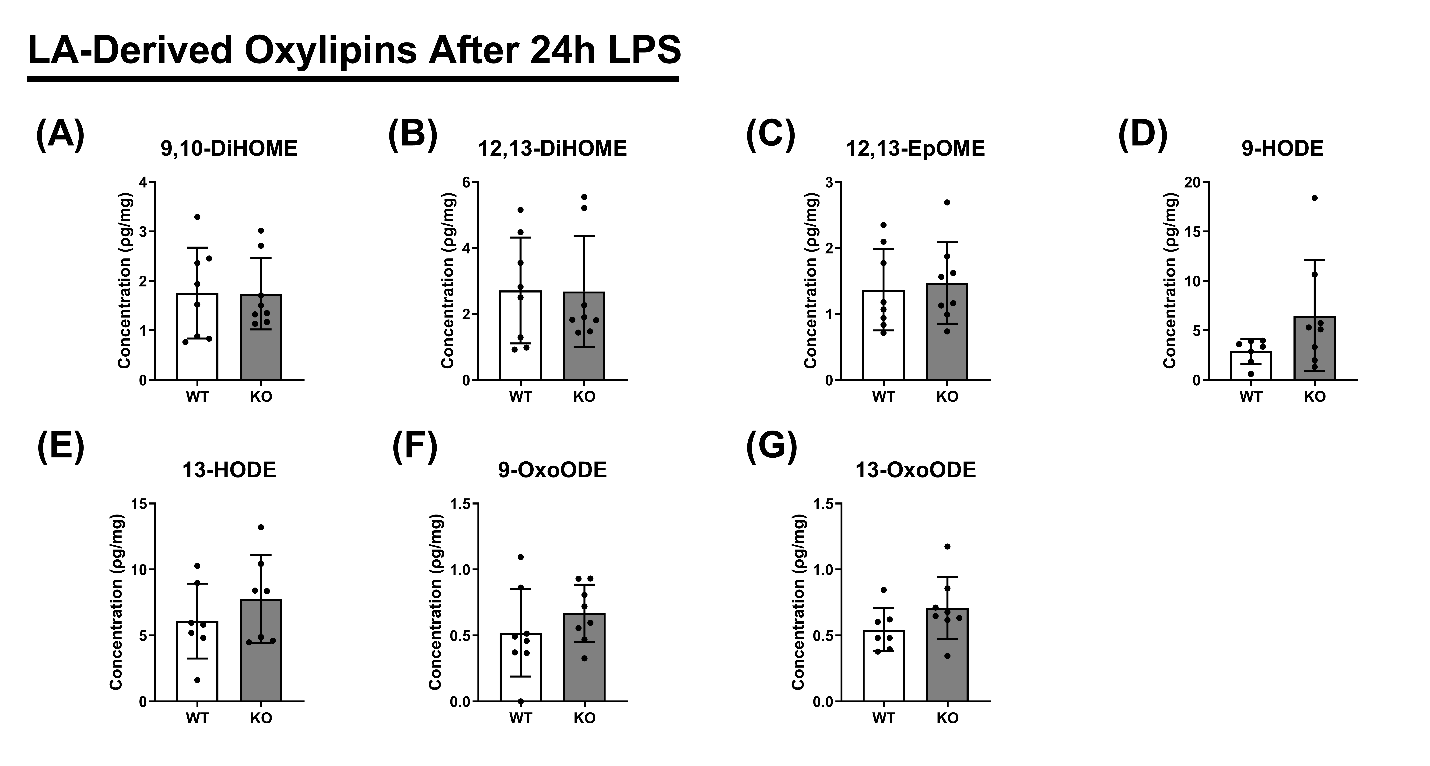


**Figure S6: The concentration of LA-derived oxylipins is unchanged after 24 h of LPS-induced acute lung injury.** The linoleic acid (LA)-derived oxylipins are **(A)** 9,10-dihydroxyoctadecenoic acid (9,10-DiHOME), **(B)** 12,13-dihydroxyoctadecenoic acid (12,13-DiHOME), **(C)** 9,10-epoxyoctadecenoic acid (9,10-EpOME) **(D)** 12,13-epoxyoctadecenoic acid (12,13-EpOME), **(E)** 9-hydroxyoctadecenoic acid (9-HODE), **(F)** 13-hydroxyoctadecenoic acid (13-HODE), **(G)** 13-oxooctadecenoic acid (13-OxoODE), **(H)** 13-oxooctadecenoic acid (13-OxoODE). Left lungs from mice aged 21–22 weeks were used for targeted LC-MS/MS analysis. Data are mean ± SD (n = 7-8) from one experiment.
